# Effects of a higher dose of alglucosidase alfa on ventilator-free survival and motor outcome in classic infantile Pompe disease: an open-label single-center study

**DOI:** 10.1007/s10545-015-9912-y

**Published:** 2016-01-14

**Authors:** C. M. van Gelder, E. Poelman, I. Plug, M. Hoogeveen-Westerveld, N. A. M. E. van der Beek, A. J. J. Reuser, A. T. van der Ploeg

**Affiliations:** 1 Department of Pediatrics, Division of Metabolic Diseases and Genetics, Center for Lysosomal and Metabolic Diseases, Erasmus MC University Medical Center, Dr Molewaterplein 60, 3015 GJ Rotterdam, The Netherlands; 2 Department of Clinical Genetics, Center for Lysosomal and Metabolic Diseases, Erasmus MC University Medical Center, Rotterdam, The Netherlands; 3 Department of Neurology, Center for Lysosomal and Metabolic Diseases, Erasmus MC University Medical Center, Rotterdam, The Netherlands

## Abstract

**Background:**

Though enzyme-replacement therapy (ERT) with alglucosidase alfa has significantly improved the prospects for patients with classic infantile Pompe disease, some 50 % of treated infants do not survive ventilator-free beyond the age of 3 years. We investigated whether higher and more frequent dosing of alglucosidase alfa improves outcome.

**Methods:**

Eight cross-reactive immunological material (CRIM) positive patients were included in the study. All had fully deleterious mutations in both *GAA* alleles. Four received a dose of 20 mg/kg every other week (eow) and four received 40 mg/kg/week. Survival, ventilator-free survival, left-ventricular mass index (LVMI), motor outcome, infusion-associated reactions (IARs), and antibody formation were evaluated.

**Results:**

All eight patients were alive at study end, seven of them remained ventilator-free. The patient who became ventilator dependent was treated with 20 mg/kg eow. Three of the four patients receiving 20 mg/kg eow learned to walk; two of them maintained this ability at study end. All four patients receiving 40 mg/kg/week acquired and maintained the ability to walk at study end (ages of 3.3–5.6 years), even though their baseline motor functioning was poorer. There were no apparent differences between the two dose groups with respect to the effect of ERT on LVMI, the number of IARs and antibody formation.

**Conclusions:**

Our data may suggest that a dose of 40 mg/kg/week improves outcome of CRIM positive patients over that brought by the currently recommended dose of 20 mg/kg eow. Larger studies are needed to draw definite conclusions.

## Background

Pompe disease (glycogen storage disease type II, OMIM #232300) is a rare, autosomal recessive lysosomal storage disorder caused by deficiency of acid α-glucosidase and characterized by lysosomal glycogen storage, mainly in muscle tissue (Hirschhorn and Reuser 2001). Depending largely on how much enzyme activity is preserved, it can present at different ages, from soon after birth to late adulthood. Patients with the classic infantile form present in the first months of life with generalized muscle weakness, hypertrophic cardiomyopathy, respiratory problems, and feeding difficulties (van den Hout et al 2003). If untreated, they usually die before one year of age due to cardio-respiratory insufficiency.

Patients’ prospects were significantly improved in 2006, when enzyme-replacement therapy (ERT) with recombinant human acid α-glucosidase (Myozyme®, alglucosidase alfa) was approved. ERT prolongs lifespan, improves cardiac hypertrophy, and enables patients to reach previously unmet motor milestones (Van den Hout et al 2000, 2004; Kishnani et al 2007, 2009; Chakrapani et al 2010; Hahn et al 2015). However, response to treatment varies between patients. When treated with either 20 or 40 mg/kg every other week (eow), approximately half of patients with classic infantile Pompe disease do not survive ventilator-free beyond the age of 3 years (Kishnani et al 2009). Similarly, a substantial proportion of patients do not learn to walk, and nearly all retain residual muscle weakness (Muller et al 2009; Case et al 2012; van Gelder et al 2012). Effective clearance of glycogen from skeletal muscle is reported in only a small number of patients (Winkel et al 2003; Van den Hout et al 2004; Thurberg et al 2006; Kishnani et al 2007, 2009). Preclinical studies in mice (Bijvoet et al 1999; Raben et al 2003) and clinical studies in infantile patients (Van den Hout et al 2000, 2004; Kishnani et al 2007, 2009; McVie-Wylie et al 2008) have shown that the reduction in glycogen levels in skeletal muscle is dose-dependent. On the basis of these findings and of the published intracellular half-life of alpha-glucosidase (Van der Ploeg et al 1988, 1991; Kamphoven 2004; Maga et al 2013), we estimated that patients might benefit from a higher and more frequent dose. We therefore treated affected infants with a dose of 40 mg/kg/week, i.e., the dose previously administered to four infants treated with recombinant human acid α-glucosidase from rabbit milk (Van den Hout et al 2000, 2004). The safety and efficacy of this higher and more frequent dosing regimen was compared with that of the recommended dose of 20 mg/kg eow.

## Methods

### Patients

Classic infantile Pompe disease was defined as symptoms of muscle weakness within six months of birth, hypertrophic cardiomyopathy, and confirmation of total deficiency of acid α-glucosidase (GAA) activity combined with the finding of pathogenic mutations in both GAA alleles. From 2009 on we treated new patients with 40 mg/kg/week. In the current study we compared patients who started treatment with the recommended dose of 20 mg/kg eow (start before 2009) to patients who started with a dose of 40 mg/kg/week (start after 2009) and who had received the treatment for at least 3 years. Data of this ongoing investigator driven study were included until April 1 2014; or until a dose change. The study was performed independent from industry. The Medical Ethical Committee at Erasmus MC University Medical Center approved the protocols and all parents gave written informed consent.

None of the patients received immunomodulation. Only CRIM positive classic infantile patients were included, which means that the combined set of very severe mutations led to the production of at least some in-active alpha-glucosidase protein. Due to the small number of patients no comparative statistics were applied.

### Clinical efficacy

Clinical efficacy was measured by assessing survival, ventilator-free survival, number of hospitalizations for respiratory infections, cardiac dimensions, and motor function. Cardiac dimensions were measured by 2D-guided M-mode echocardiographic tracings (using a Philips iE33 xMAtrix Echocardiography System, Philips Medical Systems, Andover, MA, USA), at baseline and at regular intervals thereafter. Left-ventricular mass index (LVMI) was calculated as a measure for hypertrophic cardiomyopathy (LVMI > +2z-scores (Poutanen and Jokinen 2007)) and left ventricular internal dimension (LVID) as a measure for ventricular dilatation. Motor function was examined using the Alberta Infant Motor Scale (AIMS) (Piper and Darrah 1994) and the achievement of motor milestones was examined at regular clinical assessments.

### Safety

Safety assessments included the monitoring of infusion-associated reactions (IARs). Adverse events that were judged to be possibly, probably or definitely related to ERT were considered to be IARs. The severity of each IAR was indexed by clinical judgment as mild, moderate or severe (Van den Hout et al 2004).

Before enzyme infusions, blood samples were drawn at regular intervals to measure antibodies to ERT with an enzyme-linked immunosorbent assay (ELISA) (van Gelder et al 2014).

### Pharmacokinetic analysis

To determine the activity of acid α-glucosidase in the blood circulation and the rate of alglucosidase alfa clearance in relation to dosing, we measured the activity in plasma during enzyme infusions with 20 mg/kg and 40 mg/kg. Blood samples were drawn before the start of the infusion, at 2 and 3 h after start, at 15 min before the end, at the end of infusion, and then 15, 30, 60, and 120 min thereafter.

To determine the percentage of the enzyme in the blood that was antibody-bound, patients’ plasma samples were incubated in the presence of Protein-A Sepharose beads to bind antibody-bound alglucosidase alfa, and in parallel in the presence of Sepharose beads only (control). After removal of the beads by centrifugation, acid α-glucosidase activity was measured in the supernatant (de Vries et al 2010). Pre-infusion serum samples were collected to determine the corresponding patients’ antibody titers by ELISA (van Gelder et al 2014).

## Results

### Patients

We included eight patients with classic infantile Pompe disease, four of whom were treated with alglucosidase alfa in a dose of 20 mg/kg eow and four with 40 mg/kg/week. The patients’ characteristics are summarized in Table 1. Patients in the 20 mg/kg eow dose group started ERT at a median age of 0.9 months (range 0.1–2.2 months) vs. a median age of 3.1 months (range 0.3–4.6 months) in the 40 mg/kg/week group. The median age at study end was 4.1 years (range 1.7–9.4 years) in the 20 mg/kg eow dose group and 3.5 years (range 3.3–5.6 years) in the 40 mg/kg/week dose group. All patients had very severe mutations in the GAA gene (Table 1, www.pompecenter.nl).

**Table 1:** Patient characteristics related to infusion-associated reactions (IARs)

Patient	Gender	Age at start of ERT in months	Age at study end in months (years)	Mutation I	Mutation II	Total no. of IARs possibly related to ERT (no. severe)	ERT duration at first IAR in months (in years)	ERT duration at last IAR in months (in years)
20 mg/kg eow								
1	M	0.1	33 (2.7)^#^	c.1460 T > C	c.1460 T > C	18 (1)	2.9 (0.2)	29.5 (2.5)
2	F	0.5	113 (9.4)	c.2481 + 102_2646 + 31del	c.2481 + 102_2646 + 31del	None	NA	NA
3	M	1.2	66 (5.5)	c.1933G > T	c.525delT	27 (0)	3.2 (0.3)	19.9 (1.7)
4	M	2.2	20 (1.7)	c.2481 + 102_2646 + 31del	c.525delT	3 (1)	8.1 (0.7)	17.3 (1.4)
Total						48 (2)		
40 mg/kg/week								
5	F	0.3	42 (3.5)	c.525delT	c.1933G > A	2 (0)	1.4 (0.1)	9.4 (0.8)
6	F	2.4	67 (5.6)	c.2481 + 102_2646 + 31del	c.2481 + 102_2646 + 31del	70 (6)	0.7 (0.1)	37.6 (3.1)
7	M	3.8	39 (3.3)	c.2481 + 102_2646 + 31del	c.525delT	10 (0)	0.9 (0.1)	10.3 (0.9)
8	F	4.6	41 (3.4)	c.378_379del	c.2104C > T	5 (0)	9.7 (0.8)	12.2 (1.0)
Total						87 (6)		

*M* male; *F* female; *eow* every other week; *IAR* infusion-associated reaction; *ERT* enzyme-replacement therapy, *NA* not applicable

^a^Patient developed respiratory insufficiency

### Clinical efficacy

#### Survival and ventilator-free survival

At baseline, four of the eight patients required supplemental oxygen; 50 % in both dose groups. Oxygen supply was discontinued in all patients within months after start of treatment.

At study end, one of the four patients in the 20 mg/kg eow dose group had developed respiratory insufficiency and became ventilator dependent at the age of 2.7 years during a pneumonia incident (Table 1). In the 40 mg/kg/week group none had developed respiratory insufficiency. All patients are alive.

#### Hospital admissions for respiratory infections

After the start of ERT, three of the four patients treated with 20 mg/kg eow were repeatedly hospitalized for respiratory infections or aspiration pneumonias, the number of admissions ranged from 3 to 5. In the 40 mg/kg/week group none of the patients were admitted for respiratory infections or aspiration pneumonias since the start of ERT and all were discharged from hospital within 3 weeks after the start of ERT.

#### Cardiac outcome

**Fig. 1:**
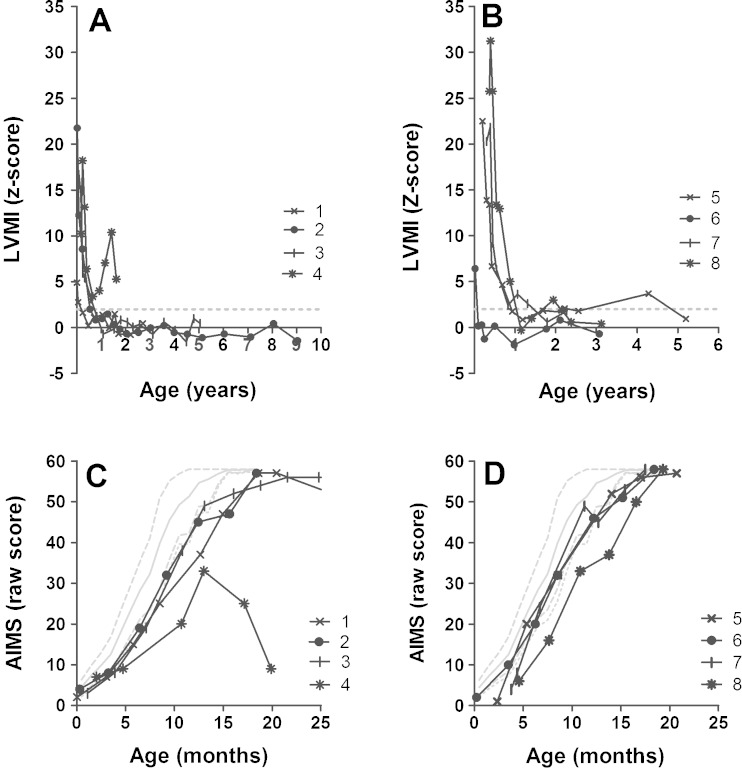
Left-ventricular mass index (LVMI) z-scores and Alberta infant motor scale scores over time. The different symbols represent different patients. LVMI 20 mg/kg eow (**a**) and 40 mg/kg/week group (**b**); The *dashed gray line* represents the upper limit of normal (+2 z-scores). AIMS 20 mg/kg eow (**c**) and 40 mg/kg/week group (**d**). The *different symbols* represent different patients. *Gray solid line*: p50; *dashed gray line*: p90 and p10; *dotted gray line*: p5

Median baseline LVMI was similar in both groups; in the 20 mg/kg eow group (median z-score +13.5, range z-score 4.9–21.8) and in the 40 mg/kg/week dose group (median z-score +21.4, range z-score 6.4–25.8). LVMI steadily decreased in both dose groups (Fig. 1a, b). At study end, LVMI was within normal limits in three of four patients in the 20 mg/kg eow dose group and in all patients in the 40 mg/kg/week dose group.

Important to note is that one of the patients, treated with 40 mg/kg/week, had severe left-ventricular dilatation and severe mitral valve regurgitation at baseline, which was considered to be life threatening by the treating cardiologist. After 1.6 years of treatment this patient’s LVMI had normalized and mitral regurgitation had become moderate. End-diastolic left ventricular internal dimension (LVIDd) and shortening fraction had also become normal (Kampmann et al 2000; Park 2008).

#### Motor function

At baseline, all eight patients showed symptoms of muscle weakness, including head lag and axial hypotonia; six had AIMS scores below the 5th percentile (2/4 in the 20 mg/kg eow and 4/4 in the 40 mg/kg/week dose groups (Fig. 1c, d)). During treatment, seven of the eight patients ultimately approached the maximal AIMS score and learned to walk: 3/4 in the 20 mg/kg eow dose group (median age at walking 16 months, range 15–17 months), and 4/4 in the 40 mg/kg/week dose group (median age at walking 15 months, range 14–17 months). Over time, some patients lost motor milestones (Fig. 1c). One patient who had initially learned to walk lost this skill after becoming ventilator-dependent at the age of 2.7 years. The only patient who did not learn to walk temporarily lost the ability to attain a sitting position after a respiratory syncytial virus infection at the age of 1.3 years. The loss of motor milestones was observed only in the 20 mg/kg eow group and not in the 40 mg/kg/week dose group. At study end, two of the four patients in the 20 mg/kg eow group were able to walk compared to all four patients in the 40 mg/kg/week group. Yet, muscular problems such as facial-muscle weakness, weakness of the neck flexors, and ankle dorsiflexors weakness were observed in patients treated with 40 mg/kg/week.

### Safety

#### Infusion-associated reactions

IARs were experienced by 3/4 patients treated with 20 mg/kg eow and by 4/4 patients treated with 40 mg/kg/week (Table 1). The number of IARs per patient varied substantially. One patient in the 40 mg/kg/week dose group had 70 IARs (over 50 % of all IARs), six of them were severe. Remarkably, the IARs started within minutes of the start of the infusion, when the infusion rate was still slow. Total IgE, serum tryptase, and complement levels were within the normal range. Two patients treated with 20 mg/kg eow had one severe IAR each. The most common IARs were exanthema, fever, and decreased oxygen saturation. All IARs could be controlled by slowing the infusion rates and prolonging the duration of the infusion, with or without the administration of antihistamines and/or steroids. No patients discontinued treatment because of IARs, all recovered without sequelae, and premedication could be stopped. At the end of the study, 4/4 patients treated with 40 mg/kg/week had been IAR-free for at least 1.5 years and all received infusions at home.

#### Antibody formation

**Fig. 2:**
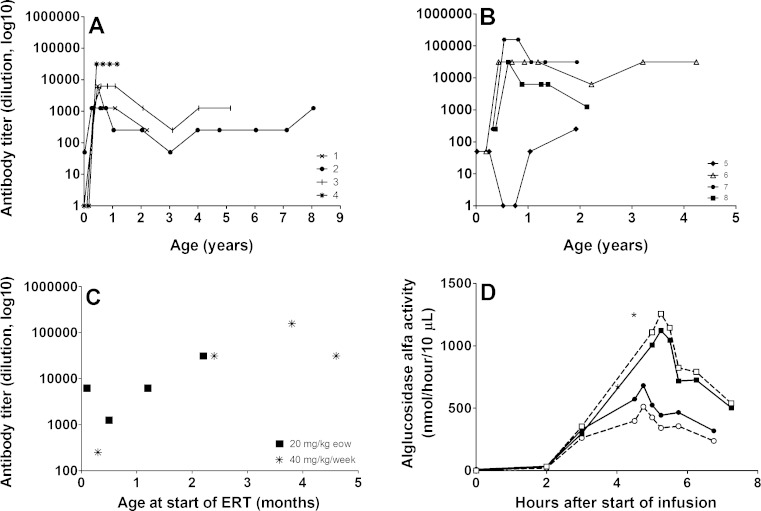
Antibody titers to alglucosidase alfa and enzyme activity in plasma using doses of either 20 mg/kg or 40 mg/kg. Antibody titers to alglucosidase alfa over time in 20 mg/kg eow (**a**) and 40 mg/kg/week group (**b**). Peak antibody titers in relation to age at start of ERT. Patients received either 20 mg/kg eow (*squares*) or 40 mg/kg/week (*asterisks*) (**c**). Enzyme activity (**d**): blood samples were collected just before the start of infusion (0 h) and at regular time intervals thereafter. A dose of 20 mg/kg (*circles*) and 40 mg/kg (*squares*) were given to the same patient (patient 3) at 1 week interval after 5.5 years of therapy (titer 1:6250). *Closed symbols* represent total acid α-glucosidase activity in the plasma; *open symbols* represent the amount of activity that was not antibody-bound. The activity in the supernatant was measured with MUGlc and is expressed in nmol 4 MU liberated per 10 μl supernatant per hour. NB: Even though the enzyme-activity assay is a standardized and validated assay there is always a slight variation in the figures obtained. All samples were analyzed as part of one experiment. The total set of analyses were performed three times with comparable results

Figure 2a, b shows the antibody titers to alglucosidase alfa of the two groups over the entire study period. In the 20 mg/kg eow dose group the median peak antibody titer was 1:6250 (range 1:1250–1:31,250); in the 40 mg/kg/week dose group the median peak was 1:31,250 (range 1:250–1:156,250).

Peak antibody titers of patients who started ERT before the age of 2 months ranged from 1:50–1:6250, those of patients who started ERT later ranged from 1:31,250–1:156,250 (Fig. 2c).

### Pharmacokinetic profile

We studied differences in the pharmacokinetics of alglucosidase alfa administrations of 20 mg/kg and 40 mg/kg by giving both doses to the same patient at an interval of 1 week. A 40 mg/kg infusion led to approximately twice the enzyme activity in plasma as compared to a 20 mg/kg infusion (Fig. 2d). The plasma half-life seemed independent of the dose.

Around the time that these experiments were performed, this patient’s antibody titer was 1:6250. Using a Protein-A Sepharose based precipitation method, we could not detect substantial amounts of antibody-bound alglucosidase alfa during enzyme infusion (Fig. 2d). Neither could we detect antibody-bound alglucosidase alfa in the plasma of three other patients who received 40 mg/kg/week and had antibody titers ranging from 1:1250 to 1:31,250 (patients 6, 7, and 8) at the time of investigation.

### Dose increase at time of clinical deterioration

In three of the four patients in the 20 mg/kg eow dose group the dose was increased to 40 mg/kg/week (ages 1.7, 2.7 and 5.5 years). This decision was made because the patients experienced life threatening respiratory infections leading to respiratory insufficiency in one of them.

After dose increase, respiratory infections disappeared in two patients. In the third patient, who had become ventilator dependent, ventilation remained required in supine position and during respiratory infections during the day. The two patients who were not able to walk did not regain walking ability. Patients are all alive 5 years after dose increase.

## Discussion

It is unquestionable that the introduction of enzyme replacement therapy has significantly improved the life expectancy of patients with classic infantile Pompe disease (Van den Hout et al 2000, 2004; Kishnani et al 2007, 2009; Chakrapani et al 2010). Nevertheless, nearly 50 % of the infants treated do not survive ventilator-free (Kishnani et al 2007, 2009). In this study we evaluated the efficacy and safety of a higher and more frequent dosing regimen, which we hoped would improve the patients’ clinical outcome.

Preclinical studies in mice have shown a dose dependent uptake of alglucosidase alfa in the range from 10 to 100 mg/kg (Raben et al 2003; Kamphoven 2004; McVie-Wylie et al 2008; Khanna et al 2012). In the very first clinical study in which we treated classic infantile patients with recombinant human alpha-glucosidase from rabbit milk, we observed a similar dose dependent effect in that the alpha-glucosidase activity in the skeletal muscle only normalized when the dose was increased from 15 to 20 mg/kg/week to 40 mg/kg/week (Van den Hout et al 2000, 2004, Winkel et al 2003). It is known that muscle cells are hard to treat since only a small fraction of infused enzyme actually reaches the muscle cells. Further it is by now generally accepted that treatment needs to be started before irreversible muscle damage has occurred. This combined experience was reason for us to treat patients with a dose of 40 mg/kg/week from start and not to wait until patients deteriorated. Earlier no difference in clinical response was found between infantile patients treated with either 20 mg/kg/eow and 40 mg/kg/eow (Kishnani et al 2009). This might be attributed to the lower dose and larger dose interval. Another factor that may have played a role is that there were more CRIM negative patients in the higher dose group (Kishnani et al 2009; Banugaria et al 2011, van Gelder et al 2014). CRIM negative patients tend to perform poorer. We therefore excluded CRIM negative patients from the current study.

The most notable contrast we observed between the two dose groups was the difference in overall clinical condition, which was reflected in the difference in hospital admissions for the two groups: while none of the patients treated with 40 mg/kg/week had ever had respiratory infections requiring hospitalization, 3/4 patients treated with 20 mg/kg eow required frequent readmissions. Consequently, one of these patients developed respiratory insufficiency at the age of 2.7 years. Our study results suggest that the 40 mg/kg/week dosing regimen helps to stabilize or improve the respiratory condition of affected infants better. Similarly, motor function appeared to be better in the 40 mg/kg/week dose group, all of whom learned to walk and maintained the ability to do so. Unlike 3/4 patients treated with 20 mg/kg eow learned to walk and only 2/4 could still walk at the end of the study. The loss of motor milestones in the 20 mg/kg eow dose group was preceded by infections requiring hospital admissions. Importantly walking was not regained in our patients when the dose was increased after deterioration. Recently two studies also reported minor effects of dose increase when patients perform poorly (Case et al 2015; Hahn et al 2015). It should also be noted that response to ERT varies between patients treated with the same dose. This is illustrated by 1/4 patients treated with the lower dose of 20 mg/kg eow, who performed well until the end of the follow-up at the age of 9 years.

With regard to cardiac hypertrophy, both dosing regimens worked equally well, which is explained by the fact that a lower dose is required to correct or prevent the cardiac hypertrophy compared to the skeletal muscle weakness (Bijvoet et al 1999; Van den Hout et al 2000; Raben et al 2003). For the same reason, adults with Pompe disease with residual α-glucosidase activities of up to 25 % do not generally develop hypertrophic cardiomyopathy, while they do have skeletal muscle weakness (Hirschhorn and Reuser 2001).

Although we observed no clear differences in safety parameters, the small numbers do not allow us to draw firm conclusions. While nearly all patients in each dose group experienced IARs the overall number of IARs was higher in the 40 mg/kg/week dose group. This was largely due to a single patient that had had over 50 % of the total number of IARs. A similar pattern was observed in the pivotal trials (Kishnani et al 2007, 2009). The patient with most IARs in our study had recurrent episodes of exanthema, coughing and vomiting, occasionally accompanied by saturation drops. Remarkably, the IARs started within minutes of the start of the infusion, when the infusion rate was still slow. Total IgE, serum tryptase and complement levels were within the normal range. While this patient had a relatively high sustained antibody titer, the titer was similar to that of other patients who did not develop as many IARs. At the time of writing, the patient was receiving home-based enzyme therapy without problems, and time since last IAR was over 2 years.

It is well recognized that therapeutic proteins can induce an immunological response that neutralizes the effect of ERT. Three of the four patients treated with 40 mg/kg/week and two of the four treated with 20 mg/kg eow developed a peak antibody titer of 31,250 which was estimated to be the highest titer without significant consequences for ERT at a dose of 40 mg/kg (van Gelder et al 2014). Using pharmacokinetic studies in the present study, we could not detect substantial amounts of antibody-bound alglucosidase alfa during enzyme infusion in patients whose antibody titers ranged from 1:6250 to 1:31,250. One patient receiving 40 mg/kg/week had a peak antibody titer of 1:156,250, which later declined to 1:31,250. According to earlier estimates, as much as 54 % of the administered enzyme (about 10 mg/kg) is antibody-bound at a dose of 20 mg/kg and a titer of 1:156,250 (van Gelder et al 2014). If a similar amount (10 mg/kg) were bound upon administration of 40 mg/kg, about 30 mg/kg would theoretically still be available for uptake in the target tissues.

Overall, we found no apparent correlation between the level of antibodies and the dose of ERT, although patients treated with 40 mg/kg/week tended to develop higher antibody titers than those receiving 20 mg/kg eow. This is consistent with a previous study that compared the level of antibody titers between patients treated with 20 or 40 mg/kg eow (Banugaria et al 2011). In line with previous observations (Khallaf et al 2013; van Gelder et al 2014) the patient's peak antibody titer seemed to be related to the age at start of therapy.

A further point that requires attention is that muscular problems were still observed in patients treated with 40 mg/kg/week. This may be due to insufficient glycogen clearance. As glycogen also accumulates in neural tissues, including motor neurons of the spinal cord and peripheral nerves (Gambetti et al 1971), we cannot exclude the possibility that neurological damage plays a role as well.

Our study describes a limited number of CRIM positive patients. We have chosen to report on children who received at least 3 years ERT in a dose of 40 mg/kg/week. Inclusion of more patients and longer follow-up will be needed to get the full picture. So far our group of CRIM positive children starting on 40 mg/kg/week seems to have a better outcome than those who started on 20 mg/kg eow. Our data suggest that the 40 mg/kg/week has the best effect when applied from the start.

## Abbreviations

Eow, every other week; LVMI, left-ventricular mass index; IAR, infusion-associated reaction; ERT, enzyme-replacement therapy; GAA, acid α-glucosidase; AIMS, Alberta Infant Motor Scale; ELISA, enzyme-linked immunosorbent assay; MUGlc, 4-methylumbelliferyl-α-D-glucopyranoside; MU, 4-methylumbelliferon; CRIM, cross-reactive immunological material.
